# A second case of pericardial mesothelioma mimicking systemic lupus erythematosus in the literature in over 30 years: a case report

**DOI:** 10.1186/s13256-017-1237-z

**Published:** 2017-03-29

**Authors:** Carolina Mensi, Alessandro Romano, Alvise Berti, Roberto Dore, Luciano Riboldi

**Affiliations:** 1 Department of Preventive Medicine, Fondazione IRCCS Ca’ Granda-Ospedale Maggiore Policlinico, via San Barnaba 8, 20122 Milan, Italy; 2 School of Occupational Medicine, Department of Clinical Sciences and Community Health, Università degli Studi di Milano, via San Barnaba 8, 20122 Milan, Italy; 3 Department of Allergy and Clinical Immunology, San Raffaele Scientific Institute, via Olgettina 60, 20132 Milan, Italy; 4 Institute of Radiology, Fondazione IRCCS Policlinico San Matteo, Piazzale Golgi, 27100 Pavia, Italy

**Keywords:** Pericardial mesothelioma, SLE, Pericardial effusion, Pericarditis, Asbestos, Case report

## Abstract

**Background:**

Mesothelioma is a rare neoplasm which commonly develops in the pleura of people exposed to asbestos. Pericardial mesothelioma accounts for only 0.7 % of all malignant mesotheliomas and it usually presents with pericardial effusion, mimicking serositis. To date, there are approximately 200 cases of pericardial mesothelioma described in the medical literature, and little knowledge exists about the systemic manifestations of this pathology. The first and only described case of pericardial mesothelioma with autoimmune features dates back to 1984 and, in our case report, we describe the second.

**Case presentation:**

We report a case of a 45-year-old white woman whose pericardial mesothelioma was initially misdiagnosed as pericardial involvement of an autoimmune disease (systemic lupus erythematosus). After several relapses of pericardial effusion, a computed tomography scan and a biopsy with histological analysis were performed revealing neoplastic growth.

**Conclusions:**

We describe a rare case of pericardial mesothelioma in a patient with a clinical presentation compatible with lupus serositis. Clinicians should consider malignant mesothelioma in the differential diagnosis of pericardial effusion, especially when it is recurrent and not clearly explained by other causes. Cytological samples should always be obtained and, if imaging tools are suggestive for solid processes, histological confirmation is mandatory.

## Background

Pericardial mesothelioma (PM) is a very rare neoplastic entity which accounts for only 0.7 % of all malignant mesotheliomas (MMs) [1]. It is characterized by a very high mortality rate, since it develops in the pericardium and can manifest with relapsing pericardial effusion and heart tamponade thus leading to acute hearth failure. Besides, diagnosis is often made at later stages [2, 3]. Asbestos is considered to be among the principal risk factors of developing MM, especially when serous membranes are involved [1]. There are three main histopathological pictures of malignant PM: the epithelial, the biphasic, and the sarcomatoid histotypes. Epithelial and biphasic histotypes are more common than the third histotype, accounting together for approximately 75 to 80 % of the total of MM cases [3]. As regards treatment options, surgery is recommended at earliest stages while radiotherapy and chemotherapy can be considered in later stage for palliative purposes [4]. To date, approximately 200 cases of PM have been described in the medical literature, and little knowledge exists about the systemic manifestations of this pathology. The first and only described case of PM with autoimmune features dates back to 1984 [5] and, in our case report, we describe the second. The relevance of this report is to inform clinicians about the rare presentation of this neoplastic condition with symptoms and signs – that is, serositis and pericardial effusion – common to other inflammatory conditions and with systemic manifestations. Albeit rare, clinicians should remember neoplasm and, in particular, PM in those cases in which pericardial effusion is recurrent and not clearly explained by other causes.

## Case presentation

Here we report the case of a 45-year-old white woman, a non-tobacco smoker, affected by PM with systemic autoimmune manifestations.

### First hospitalization

In 2011 she was hospitalized. On examination during admission, she was afebrile, dyspneic, especially mildly orthopneic, and tachycardic while her other vital signs were normal. Her body mass index (BMI) was in the normal range. No preferential decubitus was noticed and no peculiar friction rub was heard using the stethoscope.

#### Laboratory tests

Her blood tests and blood cell count were unremarkable except for an above normal erythrocyte sedimentation rate (ESR) and mild anemia of chronic disease (Table 1). She later underwent a blood sample for antibodies to coxsackievirus, echovirus, cytomegalovirus, and Epstein–Barr virus, which were all negative, thus ruling out a possible viral infection.

**Table 1:** Anthropometric measures and laboratory test results during the hospitalizations and clinical evaluations between 2011 and 2014

Findings	2011 Hospitalization	Data from other hospital admissions	2014 Hospitalization
Height (cm)	161	–	161
Weight (kg)	56	–	49
Body mass index (BMI, Kg/m^2^) [18.5–24.99]	21.6	–	18.9
Hemoglobin (g/dL) [12.0–16]	11.1	–	10.6
Serum iron (mcg/mL) [37–147]	31	–	29
Transferrin (g/dL) [0.20–0.37]	0.28	–	0.28
Ferritin (ng/mL) [11–193]	302	–	288
Erythrocyte sedimentation rate (ESR, mm/hour) [0–20]	38	28	44
C-reactive protein (CRP, mg/dL) [0–8]	0.1	0.1	0.1
Anti-nuclear antibodies (ANA)	1/320	Present, titer 1/640, homogenous pattern	–
Anti-cardiolipin antibodies (IgG; U/mL)	–	Positive, 16.2 (2012); positive, 18.7 (2013)	–
Extractable nuclear antigens (ENA)	–	Absent	–
Anti-double-stranded deoxyribonucleic acid antibodies	–	Absent	–
Anti-Smith antibodies	–	Absent	–

*Square brackets* indicate the normal levels of each variable

#### Instrumental investigations

Several instrumental investigations were performed. An electrocardiogram showed ST elevation in all leads, suggestive for acute pericarditis. Echocardiography underlined the presence of 1 cm-wide pericardial fluids between the pericardial layers, while no suspected masses were observed. Her atrial and ventricular function as well as myocardial appearance and valves were reported as normal. An X-ray of her thorax showed a moderate enlargement of the cardiac shadow. Pericarditis was treated with ibuprofen and beta-blockers with benefit.

After the first hospitalization, in the following 2 years, she developed non-erosive polyarthritis, photosensitive rash, sicca syndrome, and several episodes of pericarditis with pericardial effusion. A complete laboratory assessment was performed and disclosed antinuclear autoantibodies (ANA 1:320), weak and transient anti-cardiolipin antibody on two separate occasions (<12 weeks), while complement proteins C3/C4, extractable nuclear antigens (ENA), double-stranded (ds) deoxyribonucleic acid (DNA) and anti-Smith antibodies, were absent or within physiological ranges. Mild anemia and an above normal ESR with normal C-reactive protein (CRP) were also detected, while counts of leukocytes with differential, biochemistry panel, and anti-phospholipid antibodies (anti-cardiolipin, anti-beta2 glycoprotein I, lupus anticoagulant) were within normal range (Table 1). The presence of pericardial disease, polyarthritis, photosensitive skin rash, and positive ANA test fulfilled the classification criteria for systemic lupus erythematosus (SLE) [6].

She was treated with prednisone 25 mg/day administered orally, hydroxychloroquine 400 mg/day, and colchicine 1 mg/day without complete resolution of the pericardial disease. In the following year, she was frequently hospitalized for serositis with pericardial effusions, always accompanied by dyspnea and disease flare with articular symptoms. The first pericardial episode was successfully treated by increasing her steroids dosage. 

An ultrasound (US)-guided pericardiocentesis was performed for diagnostic purposes; cytological and cultural analysis of pericardial effusion samples resulted negative. Instead, two more episodes were treated as exacerbations of pericardial disease by increasing corticosteroid dosage with only partial recovery. Neither symptoms nor signs attributable to constrictive pericarditis were found. Of note, her ESR levels were consistently above normal, while her serum CRP concentration was constantly within the physiological range (Table 1).

### Second hospitalization for diagnostic purposes

During her hospitalization in 2014, 3 years after the first one, her vital signs were still normal, but a moderate weight loss was noticed (Table 1).

#### Radiological examinations

**Fig. 1:**
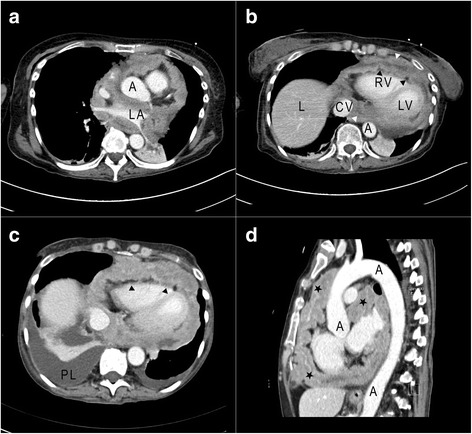
Contrast-enhanced computed tomography of the thorax before (**a**, **b**) and after (**c**, **d**) chemotherapy. **a** Axial image crossing the left atrium. **b**-**c** Axial images through the ventricles. **d** Sagittal image through the aortic arch. Before chemotherapy (**a**, **b**), both parietal (*white arrows*) and visceral (*black arrow*) layers of the pericardium were very thickened. After chemotherapy (**c**, **d**), computed tomography shows progression of the lesions around ventricles, in cranial sinuses, around the ascending aorta and the pulmonary artery. *Legend:* *A* aorta, *CV* cava vein, *L* liver, *LA* left atrium, *LV* left ventricle, *PL* pleural effusion, *RV* right ventricle, *black arrows* visceral layer of the pericardium, *star* neoplastic tissue, *white arrows* parietal layer of the pericardium

Eventually, a thorax computed tomography (CT) scan was performed and revealed extensive, irregular thickness of pericardial visceral and parietal layers, completely surrounding her heart, aortic root, pulmonary arteries, and veins (Fig. 1a, b).

#### Histological analysis

She underwent pericardiectomy with histological analysis, revealing neoplastic cell growth with immunophenotypic profiles positive for calretinin, cytokeratin chorioallantoic membrane (CAM) 5.2/cytokeratin 7/KL1, and epithelial membrane antigen (EMA), confirming epithelioid PM. According to the American Cancer Society guidelines, PM may benefit from surgery, radiotherapy, and chemotherapy [4]. Unfortunately, the PM was too advanced to be eligible for surgical removal, and the heart and aortic involvement ruled out any radiotherapy indication. She was treated with cisplatin and pemetrexed (Fig. 1c, d) without any clinical benefit and died 8 months later. Since cases of mesothelioma are subjected to special legislature in Italy and given the peculiarity of our case, a histological revision was performed by pathologists of another institute, who confirmed the diagnosis.

## Discussion

We retrospectively analyzed this case of PM in a patient who had not been exposed to asbestos who presented as having SLE with relapsing pericardial effusions, following a report to the Lombardy Mesothelioma Registry.

Mesothelioma is a rare cancer of mesothelium, which commonly develops in people exposed to asbestos. PM only accounts for 0.7 % of all MM [1]. Its association with connective tissue diseases manifesting with serositis is extremely rare. In fact, only one case of SLE as a syndrome related to PM has been described since 1980 [5]. Instead, the association of PM or peritoneal mesothelioma with inflammatory myopathies, seronegative rheumatoid arthritis, and some atypical vasculitis has been described and reported as paraneoplastic syndromes, although scleroderma-like and SLE-like syndromes (especially subacute cutaneous SLE) have also been reported [7–12].

In addition to other clinical manifestations, SLE can present with serositis, which may delay or complicate the diagnosis of PM.

In the case described, pericarditis, non-erosive arthritis, photosensitive rash, and sicca syndrome developed months before the first episode of pericardial involvement. In addition, anti-dsDNA and ENA antibodies were negative, resembling a paraneoplastic syndrome rather than a primary connective tissue disease. Although neoplasms can be commonly associated with polymyositis/dermatomyositis syndromes, the clinical and laboratory picture was not suggestive for these processes: no myalgia, no increased muscle enzymes, no specific relation with myositis-associated or myositis-specific auto-autoantibodies, and no rash typical of dermatomyositis. Moreover, the patient’s CRP levels were constantly within normal range and the poor response to corticosteroids should have suggested a cause for the relapsing pericardial effusion other than SLE, as lupus serositis tends to increase both CRP and ESR levels [13]. Besides, although her arthritis was always accompanied by pericardial effusions, it did not respond to a moderately high dose of corticosteroids, which is quite unusual for SLE.

In addition, consideration of the negative pericardial cytology for neoplastic cells and the poor response to corticosteroids should have been encouraged oriented radiological and histological approaches, even if exclusive pericardial localization of MM is particularly rare, as cytological diagnosis of MM is hindered by low sensitivity rates.

## Conclusions

In conclusion, clinicians should consider MM and in particular PM in the differential diagnosis of pericardial effusion, especially when recurrent and not clearly explained by other causes. Cytological samples should always be obtained and, if imaging tools are suggestive for solid processes, histological confirmation is mandatory.

## Abbreviations

ANA: Antinuclear autoantibodies
CRP: C-reactive protein
ds: Double-stranded
ENA: Extractable nuclear antigens
ESR: Erythrocyte sedimentation rate
MM: Malignant mesothelioma
PM: Pericardial mesothelioma
SLE: Systemic lupus erythematosus
